# Bilateral pulmonary embolism without deep venous thrombosis was observed after knee arthroscopy: a case report

**DOI:** 10.1186/s12891-021-04266-w

**Published:** 2021-04-24

**Authors:** Yuan Li, You-Xia Chen, Xiang-Tian Deng, Shun-cheng Yang, Zhi-Yuan Su, Yu-Nong Ao, Peng Zhou, Fu-Yuan Deng, Zhong Li, Jun-Cai Liu

**Affiliations:** 1 Department of Orthopaedics, the Affiliated Hospital of Southwest Medical University, Sichuan Provincial Laboratory of Orthopaedic Engineering, Luzhou, Sichuan 646000 People’s Republic of China; 2 Department of Intensive Care Unit, the Affiliated Hospital of Southwest Medical University, Luzhou, 646000 Sichuan People’s Republic of China; 3 School of Medicine, Nankai University, Tianjin, 300071 People’s Republic of China

**Keywords:** Pulmonary embolism, Knee arthroscopy, Thrombus prevention, Pulmonary embolism treatment

## Abstract

**Background:**

Symptomatic pulmonary embolism (PE) after knee arthroscopy is extremely rare. If the embolism is not treated promptly, the patient may die. Bilateral pulmonary embolism with associated pulmonary infarct without concomitant deep vein thrombosis has never been reported following routine knee arthroscopy.

**Case presentation:**

A 50-year-old female patient with no other risk factors other than hypertension, obesity, varicose veins in the ipsilateral lower extremities and elevated triglyceride (TG) presented to our ward. She had experienced sudden chest tightness, polypnea and fainting after going to the bathroom the morning of the second postoperative day and received emergency medical attention. Colour ultrasonography of the extremities showed no deep vein thrombosis. Lung computed tomography angiography (CTA) showed multiple embolisms scattered in both pulmonary artery branches. Thus, emergency interventional thrombolysis therapy was performed, followed by postoperative symptomatic treatment with drugs with thrombolytic, anticoagulant and protective activities. One week later, lung CTA showed a significant improvement in the PEs compared with those in the previous examination. Since the aetiology of PE and no obvious symptoms were discerned, the patient was discharged.

**Conclusion:**

Although knee arthroscopy is a minimally invasive and quick procedure, the risk factors for PE in the perioperative period should be considered and fully evaluated to enhance PE detection. Moreover, a timely diagnosis and effective treatment are important measures to prevent and cure PE after knee arthroscopy. Finally, clear guidelines regarding VTE thromboprophylaxis following knee arthroscopy in patients with a low risk of VTE development are needed.

## Background

Pulmonary embolism (PE) is one of the major causes of sudden death during orthopaedic perioperative care, with a mortality rate that is second only to cancer and myocardial infarction [1]. PE is common in lower limb surgery patients. Without thromboprophylaxis, the incidence of thrombotic events in total hip and knee replacement patients ranged from 29 to 60% [2, 3], and that of PE in knee arthroscopy patients was 4% [4]. There is a lack of data on the incidence of VTE in patients undergoing knee arthroscopy. Acute PE after conventional knee arthroscopy is extremely rare. Here, we report a case of successful treatment of acute PE without deep vein thrombosis (DVT) after debridement.

## Case presentation

**Fig. 1:**
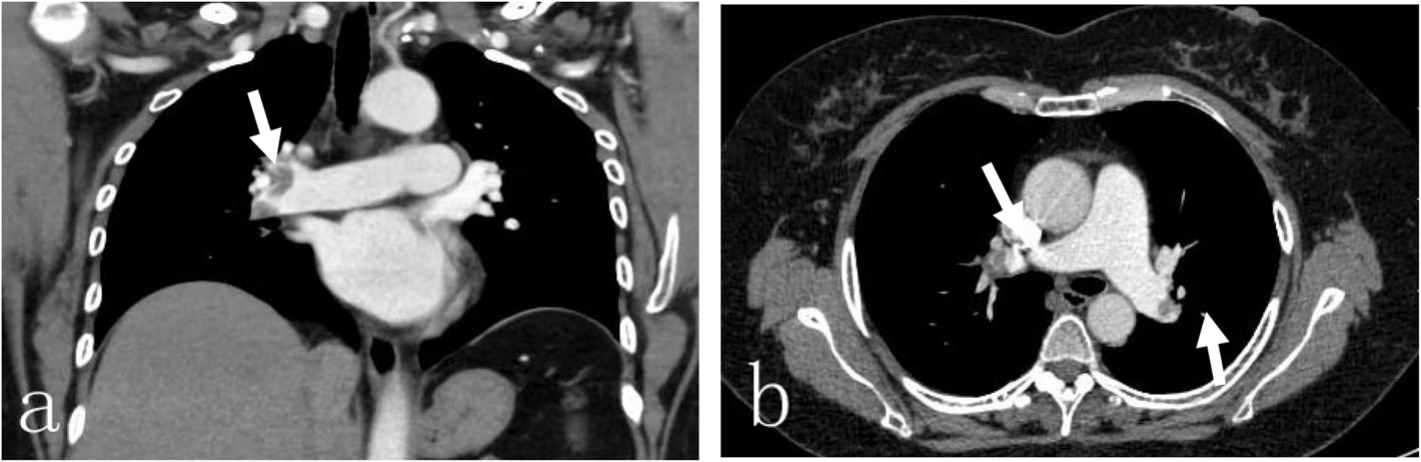
Pulmonary CTA showing multiple scattered emboli in both pulmonary artery branches, **a** coronal view showing a massive PE in the right main pulmonary artery (white arrow), **b** axial view showing a massive PEs in the left and right main pulmonary arteries (white arrow)

**Fig. 2:**
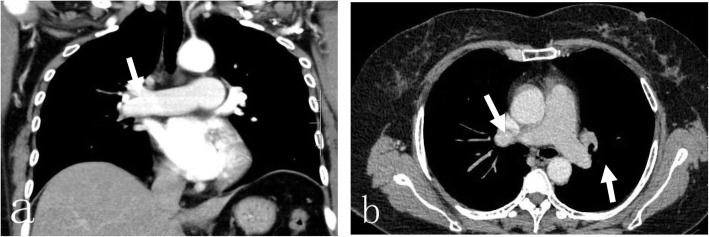
After emergency interventional thrombolytic therapy, the emboli in the pulmonary trunk and branches were significantly improved, **a** coronal view showing a small number of PEs in the right main pulmonary artery (white arrow), **b** axial view showing no significant PEs in the left and right main pulmonary arteries (white arrow)

**Fig. 3:**
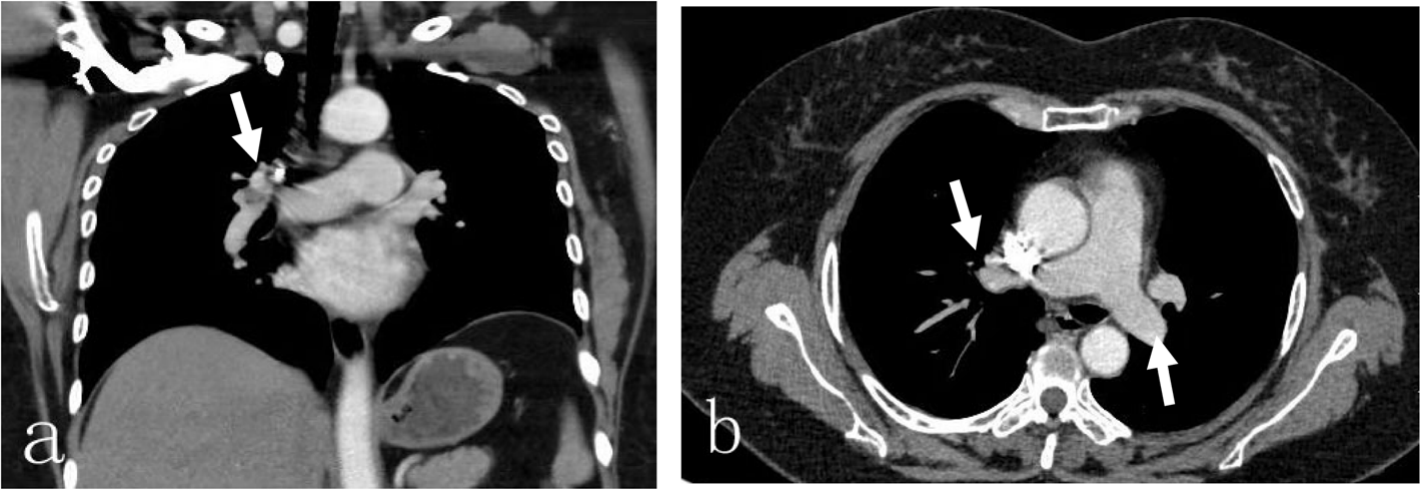
Pulmonary CTA was performed again on the 6th day after thrombolysis therapy and indicated that the pulmonary thrombi in both lungs were significantly reduced compared to those in the previous examination, with partial revascularization and clear pulmonary vascular lines, **a** coronal view showing a small number of PEs still in the right main pulmonary artery (white arrow), **b** axial view showing no new PEs in the left and right main pulmonary arteries (white arrow)

A 50-year-old female patient with an eight-plus-year history of hypertension (140–180/80–103 mmHg) and body mass index (BMI) of 36.3 kg/m^2^ was admitted to our ward due to pain during right knee joint movement for more than a year and aggravation for more than 4 months. The patient had no malignancy or venous thromboembolism (VTE) history; she had ipsilateral varicose veins and no use of anticoagulants or hormones. Magnetic resonance imaging (MRI) confirmed a degree II-III injury of the posterior horn of the medial meniscus of the right knee. On May 17, 2019, knee arthroscopy in the right knee cavity was performed under lumbar anaesthesia. The patient was operated on in a suspended-leg position. The tourniquet pressure was 260 mmHg, the perfusion pressure in the joint cavity was 70 mmHg, the tourniquet time was 25 min, and the bleeding volume was approximately 5 mL. She received postoperative fluid replenishment and pain relief and underwent physical therapy involving straight leg-raises and ankle pumping exercises. The patient did not receive any pharmacologic thromboembolic prophylaxis. She left the bed and walked on the first postoperative day, and she experienced sudden chest tightness, polypnea and fainting after going to the bathroom the morning of the second postoperative day. She received emergency medical care, including electrocardiogram (ECG) monitoring (peripheral oxygen saturation (SpO_2_): 92%, P: 98 times/min, R: 20 times/min, Bp: 103/51 mmHg). Lung auscultation revealed moist rales in both lungs. No abnormalities were detected in the blood examination (N-terminal-pro-hormone brain natriuretic peptide (NT-pro BNP): 896.40 ng/L, oxygen saturation of arterial blood (SaO2):85%, partial pressure of carbon dioxide (PCO2): 36 mmHg, D fragments of fibrin protein (D-dimer): 14.77 μg/mL, pH: 7.452, fibrin degradation product (FDP): 54.97 μg/ml, creatine kinase myocardial band (CK-MB): 7.4 μg/L, and high-sensitivity troponin T (hs -TnT): 0.16 μg/L). Doppler ultrasounds of the extremities showed normal blood flow, with no evidence of DVT or obstruction. Computed tomography (CT) angiography showed multiple scattered embolisms in both pulmonary artery branches (Fig. 1). After emergency interventional thrombolytic therapy, the emboli in the pulmonary trunk and branches was significantly improved (Fig. 2), and the symptoms of chest tightness and respiratory distress were significantly relieved, with an SpO2 of 96% and a BP of 112/75 mmHg. Postoperative medication (5 days) consisted of low-molecular weight heparin 0.6 mL ih q 12 h, 400,000 units of urokinase, and 0.9% NS 100 mL iv gtt q 12 h. After 2 h of immobilization of both lower limbs, the patient received physical therapy including knee range of motion, ankle pump and strength exercises targeting the anterior and posterior muscle groups. Pulmonary CTA was performed again on the 6th day after drug thrombolysis therapy and indicated that the pulmonary thrombi in both lungs were significantly reduced compared to those in the previous examination, with partial revascularization and clear pulmonary vascular lines (Fig. 3). The patient was discharged after evidence of improvement and received rivaroxaban 20 mg qd orally for 3 months, with monthly follow-up outpatient visits.

## Discussion

There are clear guidelines for preventing thrombus formation in the perioperative period of major orthopaedic surgery, but there is no consensus on this approach after arthroscopic surgery. Abram et al. [5] reported that the incidence of symptomatic venous thromboembolism (VTE) within 9 months after arthroscopic partial meniscal resection was approximately 0.078%. Lau et al. [6] reported that the incidence of VTE after knee arthroscopy in children was 0.27%. Some physicians believe that conventional knee arthroscopy is simple and quick, and serious complications are rare. Indeed, the risk of thrombosis is low, so there is no need for thrombosis prevention in the perioperative period [7–10]. Recently, some cases of symptomatic DVT and PTE after partial meniscus resection, anterior cruciate ligament reconstruction, multiple ligament reconstruction and repair with arthroscopy have been reported [10–12]. However, no early symptomatic PE with successful therapy after knee arthroscopy has been reported to date.

DVT is the most common cause of PE after arthroscopic knee surgery. In fact, it has been reported that 45% of patients with lower limb DVT may have PE, among which 75% of patients are asymptomatic and 4% have fatal outcomes. Without clinical PE screening, many asymptomatic patients or those with mild symptoms will not seek medical attention or will be misdiagnosed. In this case report, the patient showed no obvious abnormalities on the preoperative venous ultrasound of both lower limbs and blood examination, and the operative time was short. Although anticoagulant therapy was not used after the operation, rehabilitation via functional training and the physical prevention of lower limb DVT in both lower limbs were performed in a timely manner. This case occurred in a 50-year-old female with a BMI of 36.3 kg/m^2^, a history of hypertension for 8 years, varicose veins in the ipsilateral lower extremities, and a triglyceride (TG) level of 6.12 mmol/l; due to these characteristics, she was at high risk for DVT and should have been preoperatively treated with drugs and physical training. Recently, genetic predisposition and autoimmune antibodies have been shown to play roles in the development of DVT after knee arthroscopy. We screened for causes of spontaneous or acquired thromboembolism during outpatient follow-up [13]. The results for lupus anticoagulant, antiphospholipid syndrome, myeloproliferative disease, and paroxysmal nocturnal haemoglobinuria were negative. Unfortunately, we did not further test the levels of V Leiden, prothrombin G20210A, protein C, protein S and antithrombin [14, 15] because regardless of the outcome, this would not have changed the patient’s treatment pathway. If the patient develops venous thromboembolism again in the future, further examinations will be performed. The patient experience symptomatic PE on the second postoperative day, but no obvious DVT was found in either lower limb during vascular ultrasound re-examination. This event does not preclude the possibility of old thrombus detachment in other parts of the body or acute thrombus detachment in the affected limb. Therefore, blood vessel colour ultrasound of the extremities and even pelvic deep vein colour ultrasound should be routinely performed in patients with abnormal coagulation indicators, a genetic history of thrombosis or a high Caprini risk assessment score during knee arthroscopy to reduce the risk of thrombosis and avoid unnecessary medical disputes. In addition, we hold the opinion that more studies should be conducted to clarify risk factors for VTE development and indications for thromboprophylaxis following knee arthroscopy. Blood stasis, vascular endothelial cell injury, blood hypercoagulability, arthroscopic surgery and the presence of risk factors could lead to the development of DVT. Jiang et al. [16] considered that the use of a tourniquet for a long time could lead to ischaemia reperfusion injury to the tunica intima, thereby resulting in the formation of microthrombi. Dal Molin et al. [17] reported that continuous intraoperative arthrocentesis led to tissue oedema and pressure on peripheral veins, which subsequently led to the occurrence of DVT. Moreover, knee joint endoscopic surgery damages the inner wall of the blood vessels, slows blood flow and leads to the occurrence of DVT due to prolonged inversion, eversion and flexion of the knee joint. The stress response to surgery can activate the patient’s endogenous coagulation system, slow blood flow, and lead to the formation of DVT. Surgical trauma can induce the release of tissue factors and cytokines, activate plasminogen activator inhibitor-1, and inhibit the fibrinolytic system, eventually leading to the formation of DVT. Maletis et al. [18] suggested that in elderly patients, DVT formation occurs due to reduced daily activity, decreased muscle and vascular tension and elasticity, and medical conditions such as vascular endothelial injury and increased coagulation factor activity. A recent retrospective case-control study confirmed that patients undergoing arthroscopic knee procedures in centres at high elevations (> 4000 ft) have a 3.8-times higher risk of developing VTE than those undergoing the same procedures in centres at low elevations [19]. The patient in the current report underwent surgery at an average altitude of 1000 ft. in Luzhou, Southwest China.

Early prevention is key in reducing PE after knee arthroscopy or any orthopaedic surgery. The patient’s basic medical diseases should be actively treated before surgery, and the patient should be educated early so that they are aware of the existence and risk of thrombosis; the patient should be instructed to perform muscle contraction and diastolic exercises of the lower extremities, including ankle pump exercises and straight leg elevations. The operation should be precise to avoid injury to the blood vessels around the knee joint, and the operation and tourniquet use times should be as short as possible to reduce the risk of ischaemia reperfusion injury. Moreover, the times of knee varus and flexion should be reduced to reduce the risk of injury to the intima caused by vascular distortion. After the operation, it is necessary to closely observe the patient’s peripheral blood supply and swelling and pain in the affected limb. Laxative drugs can be given to reduce constipation, and rehabilitation training should be carried out as soon as possible.

## Conclusion

Although knee arthroscopy is minimally invasive and fast, the risk factors for PE in the perioperative period should be considered and fully evaluated to prevent pulmonary embolism. A timely diagnosis and effective treatment are important measures to prevent and cure PE after knee arthroscopy. Finally, clear guidelines regarding VTE thromboprophylaxis following knee arthroscopy in patients with a low risk of VTE development are needed.

## Data Availability

The datasets used and/or analysed during the current study are available from the corresponding author on reasonable request.

## Abbreviations

PE: Pulmonary embolism
TG: Triglyceride
CTA: Computed tomography angiography
DVT: Deep vein thrombosis
BMI: Body mass index
MRI: Magnetic resonance imaging
ECG: Electrocardiogram
SpO2: Peripheral oxygen saturation
CK-MB: Creatine kinase myocardial band
VTE: Venous thromboembolism
